# Knowledge About Postoperative Pain and Its Management in Surgical Patients

**DOI:** 10.7759/cureus.6685

**Published:** 2020-01-17

**Authors:** Muhammad Nasir, Aliya Ahmed

**Affiliations:** 1 Anaesthesiology, Aga Khan University, Karachi, PAK

**Keywords:** pain, pain management, postoperative pain, knowledge

## Abstract

Background: Research has shown that more than 50% of patients have insufficient postoperative pain relief despite the use of multiple pain management modalities. Insufficient pain relief leads to several pathophysiological effects. One of the barriers to optimal pain relief is patient’s lack of knowledge regarding the options available for pain management and their potential side effects. In this survey, we evaluated surgical patients’ knowledge about postoperative pain and its management in patients undergoing major upper abdominal surgeries at a tertiary care hospital.

Methods and Material: This was a cross-sectional survey. A total of 155 patients (18-60 years of age) scheduled to undergo elective major upper abdominal surgery were included after ethical approval and informed consent. Preoperatively, patients were interviewed through a questionnaire regarding knowledge about postoperative pain and its management.

Results: The average age of the patients was 42.97 ± 13.05 years. Excellent and good knowledge were observed in 11.61% and 21.94% patients, respectively, whereas fair and poor knowledge were seen in 42.58% and 23.87%, respectively. Inadequate knowledge was more marked regarding analgesic side effects and addiction risk. Education level, history of surgery, and adequate information provision about pain management plan by surgeons preoperatively were significantly associated with a higher level of knowledge about pain and its management (p-value 0.0005, 0.002, and 0.0005, respectively).

Conclusion: A considerable proportion of patients have inadequate knowledge about their postoperative pain and its management, particularly in areas of side effects and addiction risk.

## Introduction

A lot of research has gone into the understanding and treatment of postoperative pain in recent times, and several advances have been made in the management of postoperative pain. However, ineffective postoperative pain relief still remains a common problem in health care, and it is estimated that approximately 50%-75% of patients have insufficient pain relief postoperatively [1-3]. One of the barriers to attaining optimal pain relief has been found to be patients’ lack of knowledge regarding the options available for pain management and their potential side effects [4-8]. The reason for this is that a patient who is not aware of the available options would not be able to demand for them when in pain. On the other hand, lack of knowledge may lead to unrealistic expectations about the level of pain relief that would be achieved [6]. Thus, when working towards improvement of pain management practices, it would be useful to know the level of patients’ knowledge about pain management options and their expected side effects. The gaps identified in knowledge can subsequently be addressed by appropriate educational programs to improve patients’ knowledge. This would make patients’ expectations about the relief of his pain more realistic. Research has been conducted to assess knowledge of pain and its management in patients suffering from cancer pain to identify gaps and implement educational programs to address them [7,9,10].

Postoperative pain management is a major concern for surgical patients. Ineffective pain relief leads to several pathophysiological and psychological issues that may hinder smooth postoperative recovery [11-14]. Bozimowski has reported that patients having adequate knowledge are more likely to experience positive outcomes [6]. Furthermore, in present-day practice, patients have a right to be involved in all aspects of their pain management [14,15]. If surgical patients have adequate knowledge about the options available for postoperative pain relief, they will be better able to realistically discuss the suitable options for their situation and will also be better able to report their satisfaction and/or dissatisfaction levels [16]. To bring about improvement in patients’ knowledge regarding their pain management, the areas of lack of knowledge and the reasons behind them need to be assessed. We conducted this survey to determine the knowledge about postoperative pain and its management in patients undergoing major upper abdominal surgery at a tertiary care hospital in a developing country.

## Materials and methods

It was a cross-sectional survey and was conducted at a tertiary care hospital of a developing country. Approval was obtained from the institutional Ethics Review Committee, and informed consent was taken from all patients. Sample size was calculated by using the WHO software for sample size calculation. Previous studies have shown that knowledge about postoperative pain is approximately 70%-75% [7,9,10]. Using a value of 73%, the estimated sample size was 155 with 95% confidence interval.

Inclusion criteria to participate in the survey consisted of patients aged between 18 and 60 years scheduled to undergo elective upper abdominal surgery (incisions extending above the umbilicus). Patients who had a history of neurological or psychiatric diseases, had difficulty in communication, those unwilling to participate, and those undergoing emergency procedures were excluded. Patients coming for preoperative evaluation before major upper abdominal surgery and met our inclusion criteria were recruited. One of the authors interviewed all patients in the preoperative clinic before preoperative assessment and filled out a questionnaire. Patient’s confidentiality was ensured by assigning code numbers, and data were kept under lock and key by the primary investigator.

Questionnaire

The questionnaire for this survey was modified from the Patient Pain Knowledge Tool drafted by Borneman et al. for their work on cancer pain (with permission) and the American Pain Society Patient Outcome Questionnaire, which is permitted to be used by the Society [7,9,17]. Both these tools have been validated by the respective authors. The questionnaire included information regarding patient demographic data such as age, gender, and education level. There were 10 questions about knowledge of postoperative pain and its management. They included questions about types of pain (acute and chronic), side effects of pain medicines, risk of addiction, pain assessment scales, and treatment options other than opioids (paracetamol, non-steroidal anti-inflammatory drugs, etc.). Each question had true, false, and do not know options. The questionnaire was piloted on five patients and was presented in department academic meeting for input and suggestions before finalization. For the purpose of analysis, each correct answer was given one mark, whereas wrong and do not know options were given a zero. Patients with more than 70% correct responses were labeled as having excellent knowledge, those with 50%-69% correct responses as having good knowledge, 30%-49% correct responses were considered fair knowledge, and less than 30% as poor knowledge.

Statistical analysis

All statistical analyses were performed using Statistical Package for Social Sciences version 19 (IBM Corp., Armonk, NY). Frequency and percentages were computed for categorical observations like gender, education level, and knowledge of pain and its management. Mean and standard deviation were estimated for age. The chi-square test was applied to observe significant difference in knowledge with respect to gender, education status, and previous surgery. P-value of ≤ 0.05 was considered as significant.

## Results

A total of 155 patients were included in this survey. Overall knowledge about postoperative pain and its management was found to be excellent in 11.61% (18/155) of the study population, good in 21.94% (34/155) patients, and fair and poor knowledge were observed in 42.58% (66/155) and 23.87% of patients (37/155), respectively. Knowledge about postoperative pain and its management with respect to patients’ demographics is provided in Table 1.

**Table 1:** Knowledge about postoperative pain and its management with respect to demographic and clinical factors

Variables	Knowledge about postoperative pain and its management		Total	P-value
	Yes	No		
Age groups				0.510
≤ 31 years	11.00 (28.2%)	28.00 (71.8%)	39.0	
31-40 years	9.00 (37.5%)	15.00 (62.5%)	24.0	
41-50 years	16.00 (42.1%)	22.00 (57.9%)	38.0	
51-60 years	16.00 (29.6%)	38.00 (70.4%)	54.0	
Gender				0.104
Male	12.00 (24.5%)	37.00 (75.5%)	49.0	
Female	40.00 (37.7%)	66.00 (62.3%)	106.0	
Education level				0.0005
Illiterate	2.00 (5.6%)	34.00 (94.4%)	36.00	
High school or less	10.00 (16.9%)	49.00 (83.1%)	59.00	
Under graduate	19.00 (59.4%)	13.00 (40.6%)	32.00	
Graduate and above	21.00 (75%)	7.00 (25%)	28.00	
Previous surgery				0.002*
Yes	16.00 (59.3%)	11.00 (40.7%)	27.00	
No	36.00 (28.1%)	92.00 (71.9%)	128.00	
Received adequate information about your pain management plan from the surgeon during preoperative visit				0.0005*
Yes	22.00 (64.7%)	12.00 (35.3%)	34.00	
No	30.00 (24.8%)	91.00 (75.2%)	121.00	
Chi-square test applied. * significant.				

Age group and gender were not associated significantly with level of knowledge about postoperative pain management as shown in Table 1. Education level of the patient had a significant association with level of knowledge (p = 0.0005), patients who had received a higher education (high school and above) demonstrated more knowledge as compared to those who were less educated or illiterate. In our study, 16.8% of the patients had a history of previous surgery, and it was found that knowledge about postoperative pain management was significantly higher in patients who had a history of previous surgery (p = 0.002). Only 22% of the participants affirmed that they had received adequate information regarding pain management plans at the preoperative visits to surgery clinic, whereas 78% said that they were not provided with information about their postoperative pain management plan. Participants who received adequate information about pain management were significantly more knowledgeable as compared to those who did not receive adequate information (p = 0.0005).

Analysis of individual questions was also performed, and it was observed that most of the questions had below 50% correct response rate as shown in Table 2. The question with the highest score was related to treatment other than medicines (such as massage, heat, and relaxation) that can help relieve pain. When the question “Is the need to increase the dose of their pain medicine a sign of addiction?” was put forth, only 18.7% of the patients thought that this was not a sign of addiction, whereas 26.5% were uncertain of the answer. Only 31.6% of the patients knew about the pain scales that are used to assess pain severity. Other questions that had less than 50% correct response rates included questions evaluating patients’ knowledge regarding addiction to pain medication, prevention and treatment of their side effects, and importance for doctors and nurses to know the patients’ pain severity and characteristics as shown in Table 2.

**Table 2:** Participants’ response level to individual questions

Questions	Excellent to good response (%)	Fair to poor response (%)	Patient did not know (%)
Pain is mainly of two types: acute and chronic	61.90	22.60	15.50
Addiction to pain medicine will be caused easily when taking pain medicines	46.50	33.50	20.00
Side effects of pain medicines can be prevented and treated	28.40	48.40	23.20
It is not important for doctors and nurses to know about your pain	47.70	27.70	24.50
The pain that you are feeling can be assessed by using a pain scale	31.60	45.20	23.20
A need to increase the dose of your pain medicine is a sign of addiction	18.70	54.80	26.50
You can stop your pain medicines suddenly without worrying about side effects	21.30	46.50	32.30
Around the clock dosing of pain medicine means that the medicine will be given on regular basis, whether you are in pain or not	34.80	38.70	26.50
Paracetamol and ibuprofen are used for severe pain only	40.60	36.80	22.60
Treatment other than medicines can be helpful for relieving pain	64.50	25.20	10.30

## Discussion

This survey was conducted to assess the knowledge about postoperative pain management in patients undergoing major upper abdominal surgery at a tertiary care hospital. These surgeries were selected for the study because they cause moderate-to-severe postoperative pain that requires focused strategies including careful counseling of the patient for effective pain relief. The results revealed deficiencies in knowledge, especially pertaining to the nature of postoperative pain and the methods available to manage it. Scott and Hodson have reported similar results in their study [5]. They concluded that population of Scotland had little or no understanding of the nature of postoperative pain and methods to treat it. They recommend widespread public and professional education to make improvements to this basic clinical problem.

The results of our survey showed that only 33.55% of patients had excellent to good knowledge, whereas 66.45% had inadequate (fair to poor) knowledge regarding postoperative pain and its management following major upper abdominal surgeries. These finding are not consistent with the study conducted by Borneman et al. on patients with cancer pain [7,9]. They found that 73%-85% of the patients had sufficient knowledge regarding pain management. However, Borneman’s study was done on patients already taking pain medication. Our study, on the other hand, was conducted on a patient population who had no prior exposure to postoperative pain and its management except for about 17% of patients, who had undergone previous surgeries. Moreover, patients included in their study had a better educational background, only 18% of our study participants were university graduates whereas 23.23% were completely illiterate.

Our results did not reveal a significant difference in the level of knowledge in context to age and gender. A similar finding was established by the Norwegian study done by Valeberg . However, there was a higher percentage of female patients (68.39%) in our study compared to almost equal gender distribution in Valeberg’s study sample [18].

A significant correlation was found between educational level and the total score of knowledge about pain in our study population. The enrolled population who had graduate level education and above demonstrated better knowledge regarding pain management options than those who had received lower levels of education or were illiterate. Baker et al. found in their research that individuals with higher education actively seeked information regarding their treatment and discussed that information with their physicians, while lower education was found by them to be one of the barriers to receiving and understanding information regarding pain management [8]. Results of our survey demonstrated that patients who had previous surgical exposure had significantly better knowledge regarding postoperative pain management (p = 0.002). Scott and Hodson found a statistically signiﬁcant difference between patients who underwent previous surgery (51%) and those who did not (32%) (p = 0.001) [5]. In their study, patients who had not experienced previous surgery thought simple pain killers such as aspirin, paracetamol, or ibuprofen were sufﬁcient for major surgery, whereas others who had undergone previous surgery had knowledge about narcotic analgesia and knew that narcotics were used most often for postoperative pain management.

In our survey, patients who received adequate information about their pain management plan in the surgical clinic during the preoperative visits were more knowledgeable as compared to those who did not receive adequate information (p = 0.0005). Similar findings were observed by Stomberg et al., who concluded that inadequate knowledge about pain management was due to a lack of preoperative information and inadequate preoperative discussions about pain management [19]. They also found that inadequate preoperative discussion regarding pain management correlated with higher postoperative pain [19]. Furthermore, Miaskowski et al. found that educational intervention in cancer patients was effective in good pain management [20]. Two different studies, by Kim et al. on US population and Rustoen et al. on Norwegian population, found that patients who received psychoeducational intervention had an increased overall knowledge of cancer pain management by 21% [4,21].

More than 50% of our patients were of the belief that the need to increase the dose of their pain medicine was a sign of addiction. Borneman et al. had similar findings in their study and discovered that the belief that ‘use of opioids for pain relief leads to addiction’ was the knowledge item which had persistently low scores among their questionnaire items [9]. They found this to be an important barrier to pain management in cancer patients and recommended continued teaching and reinforcement. Their findings indicate that, although patients may have knowledge about pain and its traditional treatment, they are unaware of the adverse effects, and risk of addiction and tolerance. They recommend that these misconceptions regarding pain therapy can be alleviated through implementation of educational programs.

In our findings, the question with the highest knowledge score was related to treatments other than medicines (massage, heat, relaxation, etc.) to help relieve pain. Patients are aware of these options through various sources, such as magazine articles, family, and friends. Despite having knowledge about these strategies, patients often do not make use of them [22]. Becker et al. have identified several barriers to the use of a non-pharmacological method for pain relief [22]. These include fear that it might fail or be harmful, skepticism about its efficacy, belief that it is lower quality/substandard care, lack of motivation, and so on.

Assessment of pain is an essential part of effective pain management. The health care provider must assess patients’ pain at regular intervals to ensure that the patient is pain free or ascertain whether action needs to be taken to improve pain management. Patients’ understanding and cooperation is required for adequate pain assessment. Only 31.6% of our patients knew that pain scales are used to assess the severity of pain. Pain is a complex phenomenon that involves physical, psychological, and social variables [23]. Level of coping capacity and emotional states vary in each patient. Some patients may believe that it is noble to suffer in silence and do not inform the caregivers about their pain. It is therefore recommended that surgical patients must be informed preoperatively about the importance of pain assessment for effective pain management and the method that will be used for their pain assessment in the postoperative period should also be explained to them. This would also be an opportunity to improve their perception about the importance of doctors and nurses to know about the severity of their pain and about any side effects that they may have secondary to their medication. Adequate pain relief is essential for the patients to be able to resume activity, maintain a positive attitude, and get adequate sleep following surgery, and this requires knowledge and prioritization on the part of both doctors and nurses [24].

Bozimowski concluded that lack of knowledge about pain and its management is an important barrier to optimal pain relief [6]. Miaskowski et al. suggested that strategies in reducing barriers to pain management require attention to knowledge as well as attitudes about pain [20]. Preoperative interaction between the patient and health care providers seems to be an important tool to provide information about post-operative pain management. Clinicians can utilize patient’s responses to this type of questionnaire and focus on individualized teaching to improve this knowledge deﬁcit. Although considerable time and resources will be required to implement such an educational program, this individualized approach will eventually save staff time of physician and nurses and improve patient outcomes.

## Conclusions

The results of our survey demonstrate that patients have limited knowledge with respect to their postoperative pain management, particularly in areas of drugs, addiction risk, and pain scoring. Future research in this area should capture a larger surgical population including patients having different surgical procedures. Moreover, the impact of preoperative patient education regarding pain management practices on improvement in postoperative pain management needs to be studied.
